# Sketching the vision of the Web of Debates

**DOI:** 10.3389/frai.2023.1124045

**Published:** 2023-06-16

**Authors:** Antonis Bikakis, Giorgos Flouris, Theodore Patkos, Dimitris Plexousakis

**Affiliations:** ^1^Department of Information Studies, University College London, London, United Kingdom; ^2^Institute of Computer Science, Foundation for Research and Technology–Hellas, Heraklion, Greece

**Keywords:** online debate analysis, computational argumentation, computational persuasion, web technologies, human-centered AI

## Abstract

The exchange of comments, opinions, and arguments in blogs, forums, social media, wikis, and review websites has transformed the Web into a modern agora, a virtual place where all types of debates take place. This wealth of information remains mostly unexploited: due to its textual form, such information is difficult to automatically process and analyse in order to validate, evaluate, compare, combine with other types of information and make it actionable. Recent research in Machine Learning, Natural Language Processing, and Computational Argumentation has provided some solutions, which still cannot fully capture important aspects of online debates, such as various forms of unsound reasoning, arguments that do not follow a standard structure, information that is not explicitly expressed, and non-logical argumentation methods. Tackling these challenges would give immense added-value, as it would allow searching for, navigating through and analyzing online opinions and arguments, obtaining a better picture of the various debates for a well-intentioned user. Ultimately, it may lead to increased participation of Web users in democratic, dialogical interchange of arguments, more informed decisions by professionals and decision-makers, as well as to an easier identification of biased, misleading, or deceptive arguments. This paper presents the vision of the Web of Debates, a more human-centered version of the Web, which aims to unlock the potential of the abundance of argumentative information that currently exists online, offering its users a new generation of argument-based web services and tools that are tailored to their real needs.

## 1. Introduction

From the plain publishing of content^1^ to the collaborative contribution of knowledge through social media^2^ and the annotation of content with machine-processable semantic information,^3^ the Web has been constantly reshaping. The development of the Social Web (the social aspect of Web 2.0) has brought about a significant change in the way people use the Web. Nowadays, people around the world access the Web to rate a hotel or a restaurant; they share comments on the story and the writing style of a book; they use it to like or dislike a photograph, a video, or the whole lifework of a music band; they write opinions in blogs; they discuss subjects of any matter in forums; they substantiate opinions in wikis citing sources of diverse reliability. Currently, the Web is flooded with opinions and arguments touching topics related to just about everything important or insignificant that happens or has happened or may happen in our world.

Unfortunately, all these colorful, diverse, contradictory, interesting or indifferent opinions get lost; *scripta manent*, yet opinions are currently not uploaded as machine-processable data, they are not interlinked, and it is extremely difficult for Web users to find opinions and arguments related to a particular subject, let alone to evaluate them, characterize them based on objective or subjective criteria, or select the ones that would appeal more to them. Current search engines can only help the user access the pages containing arguments on a topic; manual effort is then required for making sense out of the multitude of contradictory and diverse results returned, for identifying the relations among the available arguments and supportive data, or for analyzing their credibility.

Building on the recent advancements in *Machine Learning, Natural Language Processing*, and *Computational Argumentation*, there have been some attempts to unlock the potential of this information. These include an ontology for representing arguments using well-defined, structured formats (Rahwan et al., 2007), methods for argument mining (Stede and Schneider, 2018; Lawrence and Reed, 2019), software tools for argument analysis and visualization (Reed et al., 2017), argument search engines (Wachsmuth et al., 2017b; Stab et al., 2018; Chen et al., 2019), persuasive chatbots (Chalaguine and Hunter, 2020), and autonomous debating systems (Slonim et al., 2021). However, existing efforts fall short in two ways: first, there is still no mature technology allowing the reliable extraction of arguments from text for annotation and further automated processing; second, there are still no general models for realistic arguments, which would be able to capture all aspects of our everyday argumentative dialogues or debates on topics of general concern, such as global warming, international politics, or the energy crisis. Especially since Dung's seminal paper on Abstract Argumentation Frameworks (Dung, 1995), we have developed a very good understanding of the relation between argumentation and logic-based reasoning. However, human dialogues and debates often involve arguments based on *implicit information* (e.g., commonsense knowledge), may resort to *unsound reasoning* (e.g., proof-by-example), or employ *non-logical argumentation methods* (e.g., peer-pressure, use of emotionally loaded arguments, authoritative claims). The study of such aspects and, more generally, the study of the *ethos* (appeal to the credibility of the speaker) and *pathos* (appeal to the emotions of the audience) of argumentation, is not yet as mature as the study of the *logos* of argumentation, in the context of Artificial Intelligence.

Furthermore, online arguments and opinions are not just put forward to be heard, but they have a *purpose* and their processing needs to be purposeful as well. There is, therefore, a need for a new generation of Web tools that will assist humans in reaching conclusions using arguments that are not only formally structured, but are also tailored to the particular characteristics of the *audience* that they are addressed to and the *context* in which they are made, in order to be better comprehensible, more relevant and, therefore, more effective. For any topic, it is important to provide Web users with an overview of all different viewpoints; it is equally important, however, the presentation of these viewpoints to take into account the background knowledge and cognitive characteristics of each individual user.

To address these challenges and needs, we propose and sketch the design of a new version of the Web, which we call the *Web of Debates*. Its ultimate goal will be to offer the means for assisting humans in participating in debates and collective decision making processes with well-justified and persuasive arguments, as well as in identifying biased, misleading or deceptive arguments. It will be a *global, human-centric AI system*, which, taking advantage of advanced AI methods, will be able to process and analyse the huge amount of natural language arguments and opinions that are available online, and provide its users with personalized, user-friendly services for retrieving, filtering, evaluating and visualizing this information, helping them better make sense of the different viewpoints, draw their own conclusions and take informed decisions about any matter of personal or public concern. The aims of this paper are to describe this vision, identify the requirements and challenges of its realization, discuss the theoretical and technological advancements that are needed to address them, and provide a roadmap toward its realization. Another aim is to demonstrate the central role argumentation can play in the development of human-centric AI systems by providing computational models and tools for cognitive reasoning and dialogues among humans and machines at the global scale. We presented some preliminary ideas on this vision in Flouris et al. (2013) and Flouris et al. (2016); here, we elaborate more on these ideas, taking into consideration the recent advancements in related fields of research such as argumentation, machine learning and natural language processing.

The not-so-distant-future example that follows illustrates how we envision the interaction with the Web of Debates (Section 2). Section 3 gives more details about the vision: it motivates the need for its realization and describes how it will function, how people will benefit from it, and its main goals. Section 4 describes the challenges that stand in the way of its realization and proposes directions to overcome them, and Section 5 discusses its potential impact and some possible ethical issues that the Web of Debates may raise. Section 6 summarizes the main points of this vision paper.

## 2. Motivating example

The day began with a feeling of unrest for Steffi. The new article she is about to prepare obtains added gravity in the prospect of her country's elections next month. The topic is not unfamiliar to her; as a financial journalist she has written numerous articles in the past regarding the financial crisis and the impact of measures suggested by the International Monetary Fund (IMF) in other countries. Her intention this time is to question the diverse viewpoints on the IMF that are put forward by the different parties and to present as objectively as possible well-justified and clearly-articulated opinions both in favor and against the controversial role of IMF.

She hits “IMF policies help countries recover from financial crises” in ArgSE, the Arguments Search Engine she mostly uses when seeking for arguments on the Web, and configures its settings in “debate mode”, in order to receive both supporting and refuting arguments. She has prepared a categorization of the different target groups she is interested in to drive the mining process, and has uploaded the corresponding profiles using the “Audience Characteristics” functionality of ArgSE. For instance, she would like to know what arguments can be more meaningful for unemployed young people and middle-class workers.

Steffi has configured ArgSE to search for relevant arguments online but ignore sources with a low credibility score. Her profile data guides ArgSE to accurately decide on the level of detail to apply for the construction and presentation of arguments: her expertise in financial terms is sufficient to understand arguments on the connection between unemployment and inflation, but those regarding certain social aspects of unemployment require more detailed analysis in order to be comprehended.

As a result, ArgSE returns a graphic showing in a visually appealing manner the different arguments, as well as their relevant properties and metadata, including the sources (provenance) of each argument, the date and time of its publication, its supporting evidence, the argument style (e.g., deductive, inductive, etc.), its adequacy for a particular audience, and the relationships among the arguments (e.g., attack, support etc). It further identifies categorizations that Steffi did not consider in the first place, classifying certain arguments to audience groups sharing similar characteristics.

Using all the available information, Steffi navigates more deeply in the graph, she filters, questions, groups and organizes the available arguments, and eventually identifies and extracts the most convincing ones. A few hours later her article is ready. Her debate-enabled editor has assisted her in annotating the different parts of her text with a formal description of the arguments they refer to, so that search engines can identify and retrieve them, and links them with the respective online sources and evidence they are based upon. Steffi's own conclusions, based on the correlation of facts she personally deduced during her research are also included (and annotated) in the text. This way, her annotated article and arguments can be stored in her electronic newspaper's argument repository for others to find and reuse. As she sends the article to her editor she feels confident that her audience will have the means to form a well-informed opinion before actively participating in the country's decision making process.

## 3. The vision of the Web of Debates

### 3.1. Why: the need for the Web of Debates

As the Web is increasingly being used for informational purposes, the public opinion is progressively being shaped by what people read online. Online versions of traditional mass media play a major role in this shift. On the other hand, due to the easiness with which content can now be uploaded, many users now use the Web as a podium to express themselves. However, extracting *meaning* out of the plethora of opinions (i.e., evaluating the credibility and coherence of information related to a subject of interest, understanding why it is important, and ultimately deciding whether to adopt or reject it) becomes increasingly difficult.

Even today's Web contains the information necessary for Steffi to complete her article. However, this information, being in textual form, is not easily retrievable or processable, so it is not appropriate for implementing the features presented in our example scenario. The Semantic Web (Berners-Lee et al., 2001) and Linked Data^4^ initiatives promised to overcome some of the limitations of natural-language Web pages by providing appropriate methodologies for publishing and interlinking semantic data on the Web using machine-processable formats. This has recently led to the development of *knowledge graphs* (graph-based representations of real-world knowledge; Hogan et al., 2021) and several types of knowledge-based systems, such as search engines, recommendation systems, personal agents, etc. However, the focus of these initiatives and models is on the representation of data, rather than arguments or opinions.

Similarly, the main tenets of computational argumentation (Besnard and Hunter, 2008; Baroni et al., 2018) and the extensive research conducted in this field have direct impact on the formulation of the new Web. This research has led to various types of applications in domains such as law, medicine, e-government and others (Atkinson et al., 2017). While they demonstrate well the potential of computational argumentation, they are all of small scale, being limited by their inability to process natural language arguments. On the other hand, the recent advancements in *argument mining* (Lawrence and Reed, 2019) have led to global-scale applications of argumentation such as argument search engines (Wachsmuth et al., 2017b; Stab et al., 2018; Chen et al., 2019). Their main functionality is to find on the Web arguments pro or con any controversial issue. *args.me* (Wachsmuth et al., 2017b) and *PerspectroScope* (Chen et al., 2019) rely on pre-structured arguments, while *ArgumenText* (Stab et al., 2018) has the ability to extract arguments from any Web document. They all rank arguments by relevance to the user-specified topic, while some of them present extra information for each argument such as supporting evidence, its stance score (denoting the extent to which it supports or refutes the claim) or its relevance score. While these are closer to the kinds of applications we envision for the Web of Debates, their performance is still limited as, for example, evidenced by the results of a recent user-based evaluation, which showed that they do not significantly outperform conventional search engines especially with respect to the *convincingness* of the arguments they retrieve (Rach et al., 2020). This can be attributed on the one hand to the limitations of the argument mining methods they use, and on the other to the lack of a method to assess the quality or persuasiveness of arguments.

Realizing the types of services and applications we describe in Section 2 requires addressing the primal reason why opinions reach the Web in the first place, which is to be *persuasive*. This latter step is important, in order to depart from simple argument listings and logical argumentation, and support *realistic arguments* and *debates with a purpose*, i.e., debates where arguments are not-purely-logical, and have a certain aim, namely to persuade a certain audience on some topic, as happens in real-world debates, or help a group of people make an informed decision through deliberation.

### 3.2. How: the function and use of the Web of Debates

**Figure 1:**
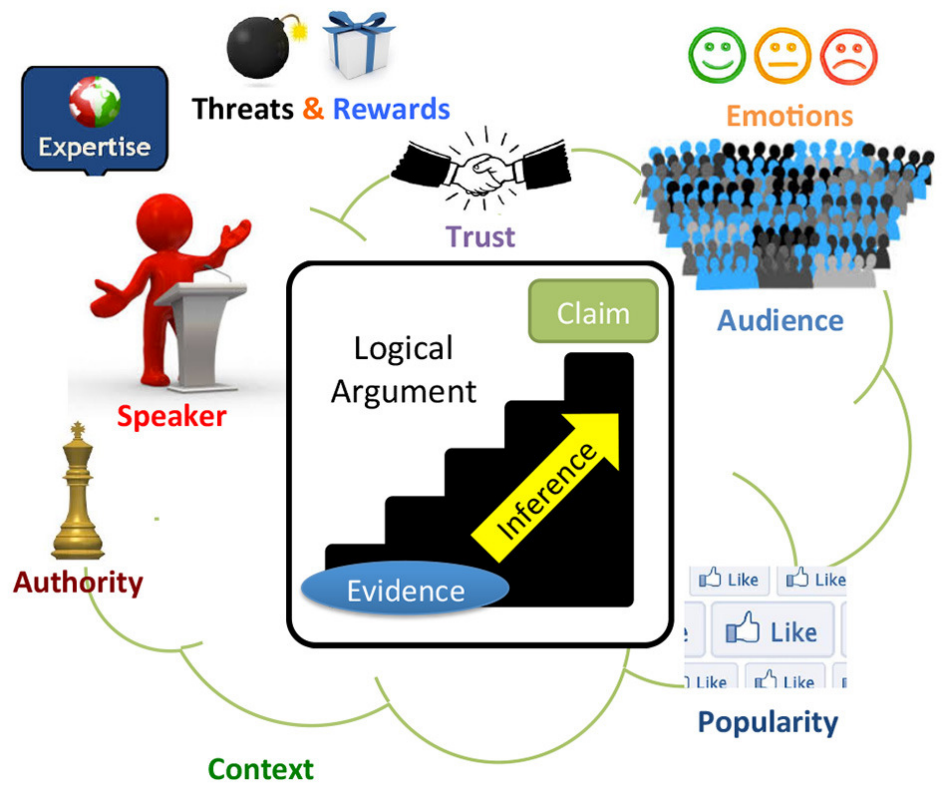
A realistic argument in the Web of Debates.

**Figure 2:**
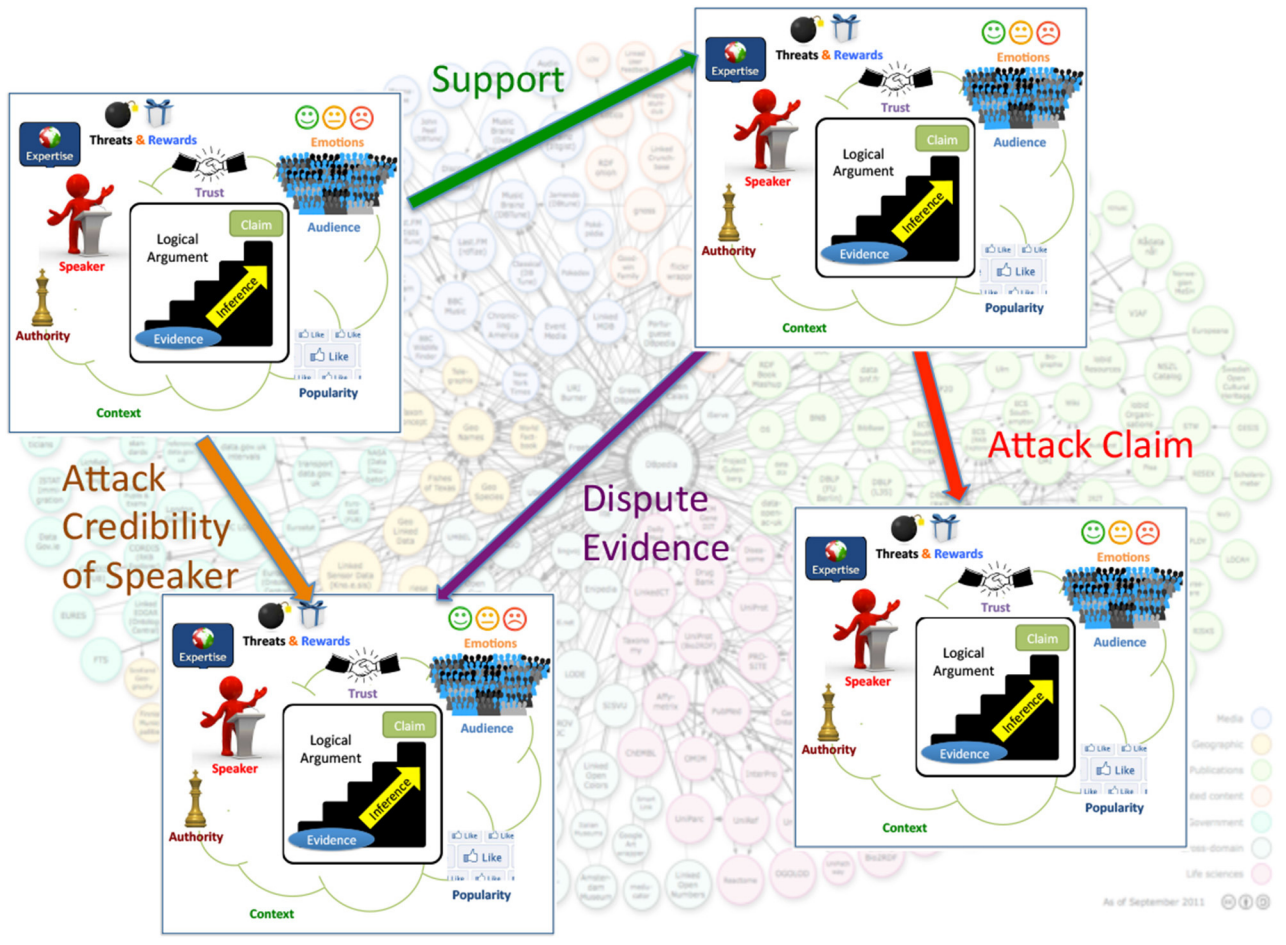
Arguments and data interplay in the Web of Debates.

Current Web technologies focus on searching for and managing documents and information. The Web of Debates will additionally enable searching for and managing *realistic arguments* (Figure 1). A realistic argument will have an internal structure, containing a logical part, but also other types of information related to its persuasiveness or general quality: the audience that it is targeted at, its provenance, the context in which it was made, the values it promotes, the popularity of the claim that it supports, evidence for its believability (e.g., links to documents, facts, or other arguments that back it up), the conditions under which it is effective or valid, etc. Moreover, arguments will be *interlinked* in various ways, where the links may represent different types of support or attack relationships among the arguments (Figure 2). Understanding the role of the different components and interconnections of realistic arguments, as well as studying the factors that affect the persuasiveness and quality of arguments, such as emotions, trust, provenance, evidence and other logical or extra-logical considerations will be a crucial first step toward realizing the vision of the Web of Debates.

The Web of Debates will revolutionize the way argumentative information that exists on the Web is organized and exploited. Arguments will be uploaded directly by content providers, but it will also be possible to construct them on demand from text or by combining existing arguments with data from knowledge graphs and other types of knowledge bases, following formal methods, and taking into account the intended audience. To allow content consumers make the most out of the presented arguments, the Web of Debates will exploit information that is both of objective nature (e.g., the structure of an argument, the logical fallacies it may contain or its relationships with other arguments) and of subjective nature (e.g., the consumer's background knowledge and cognitive characteristics), based on which a proper ranking of the presented arguments will be possible, so that the strongest, most relevant and most understandable will be more visible. This, however, will not undermine the *diversification* of the presented opinions. In order to prevent the formation of *echo chambers*^5^ or *filter bubbles*,^6^ the selection and ranking algorithms of the Web of Debates will ensure that arguments from all different viewpoints will be presented and highlighted, and the users will be allowed to access and configure the algorithms as they wish. In our motivating example, ArgSE would return the official opinions of IMF, as well as counter-opinions put forward by leading economists and other people, provided that they are trustworthy enough and understandable (per Steffi's knowledge background). It would also be able to explain how the presented arguments were selected and ranked and give the options to Steffi to configure the selection and ranking process.

Realistic arguments will be stored in “argument bases” (the analogous to knowledge bases and ontologies) and will be linked to online sources, such as a collection of sentences inside a document, information retrieved from a picture, etc. In the context of our example, people arguing about IMF's role in mitigating the effects of the economic crisis, will have the ability to post and interrelate arguments in a machine-interpretable way. Similarly, the IMF itself will be able to express its own arguments on the matter, stored in its own dedicated repository and uploaded on its website. Note that all types of digital artifacts (from financial reports to polls, simple text, images, videos, other arguments, datasets) can be used as evidence supporting a certain argument. Thus, arguments and digital objects will be interrelated in two ways: arguments can be linked to digital objects they refer to, whereas digital objects can also be used as parts of arguments (e.g., as supportive evidence).

The Web of Debates will also enable certain forms of *dialogical interaction* with its users. As described in the motivating example, after receiving a set of arguments that best match her request, Steffi will be able to follow up by requesting more arguments, by asking for more clarifying information about a certain argument, or even dispute the returned ones by presenting her own counter-arguments. ArgSE will then be able to search again in the Web of Debates and respond back by presenting additional persuasive arguments in the first case, data that back up or explain the argument in question in the second case, and data or arguments that invalidate Steffi's counter-arguments in the third case.

Summing up, we envision the Web of Debates not as a replacement for the current Web but as a complementary technology. Searching for and interlinking documents and information will still be among the core functions of the Web. The Web of Debates will provide additional tools that will exploit such functions to support a new one: the retrieval and management of arguments and the interlinking among arguments, web documents and information.

### 3.3. Who: actors in the Web of Debates

The Web of Debates will provide benefits for both the content provider and the content consumer, by offering a convenient podium for expressing one's opinions and a platform for accessing opinions of others. The easy access to the enormous amounts of Web information, in tandem with the automated annotation, retrieval, exploration and analysis of realistic arguments, will allow opinions to reach a large, literally global, audience, and, at the same time, provide a valuable tool in the hands of professionals, businesses, organizations, governments, or individuals to support their decision-making processes. This will be realized via the development of new and more powerful argument-aware search engines and other types of web applications that will allow users to retrieve, process, visualize, understand and query the arguments uploaded by content providers, as well as their interrelationships.

The combination of these features and tools will stimulate opinion diversity, contribute toward collective awareness and informed decision-making, promote active citizenship and e-democracy, support legal argumentation and justice attribution, allow improved fact-checking and encourage structured and civilized argument exchange in a networked world. In addition, it will help all parties formulate explicit opinions in their effort to persuade others into accepting a certain claim or taking a certain action, thereby using the Web to argue in favor of the products, services or ideas that they promote (for marketing or advertising purposes, or for refuting unjustified opinions or prejudices).

In our motivating scenario, Steffi is aided in her task by a graphic display summarizing the strongest arguments retrieved from credible sources on the Web, as well as their properties, supporting evidence and interrelationships. In this way, she would be protected from malicious users and sloppy arguments. Moreover, she would be able to concentrate on the most important ones or those that are most relevant to the specific context or case that she is interested in, and she would be able to easily identify poorly supported opinions.

### 3.4. What: the goal of the Web of Debates

The goal of the Web of Debates is not to impose any given opinion, but to provide the medium through which a user can “collect” different arguments in favor and/or against a certain claim in order to form an opinion of their own, convince an audience to accept a certain claim or opinion or participate in discussions with other users in order to take collective decisions about a certain course of action. The services offered by a search engine in the Web of Debates are analogous to those of a journalist, whose role is to objectively and concisely reproduce the most prominent opinions expressed by different people or entities (e.g., political parties), in ways that help the readers better understand and evaluate them, taking into account their profiles and backgrounds. In our example, ArgSE retrieves and presents arguments from sources that are considered reliable, as well as information associated to their quality and persuasive strength for audiences that match the profiles provided by Steffi. But it is up to Steffi to decide which of them would actually be the most influential for the readers of the newspaper she is working for. Apart from search engines, the Web of Debates will support several other types of applications, such as everyday assistants, expert companion systems (see e.g., Dietz et al., 2022 for some examples), collaborative decision support systems, intelligent tutoring systems aimed at teaching users how to make better arguments, automated debating systems and others. Some common characteristics of all such systems will be their focus on natural-language arguments and the human aspects of argumentation, their seamless integration within the online, private or social, activities of their users, their adaptability to background knowledge and cognitive characteristics of each user or group of users, their ability to explain any inferences they make, and their ability to develop by learning from experience and by taking into account the feedback provided by their users. In other words, they will combine all major characteristics of *human-centric* AI systems.

## 4. Realizing the vision

**Figure 3:**
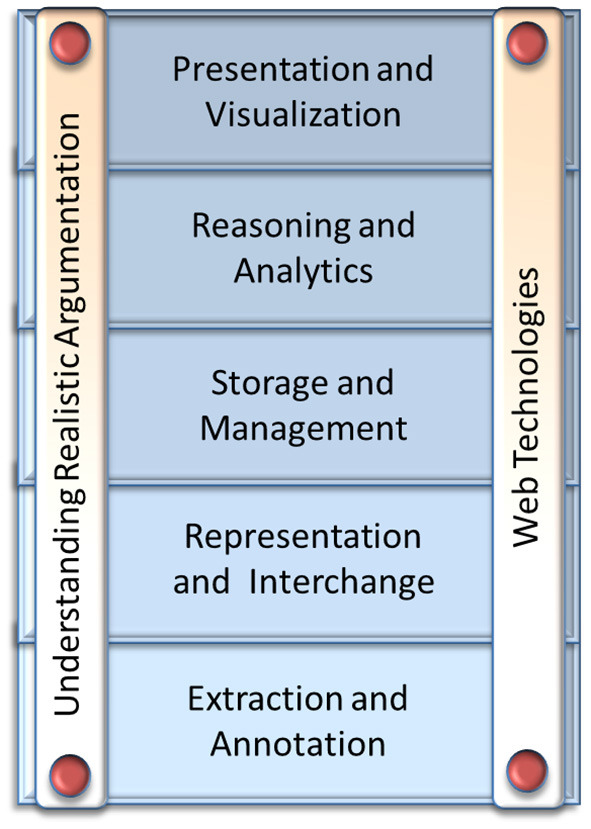
Related technologies and challenges toward the Web of Debates vision.

**Figure 4:**
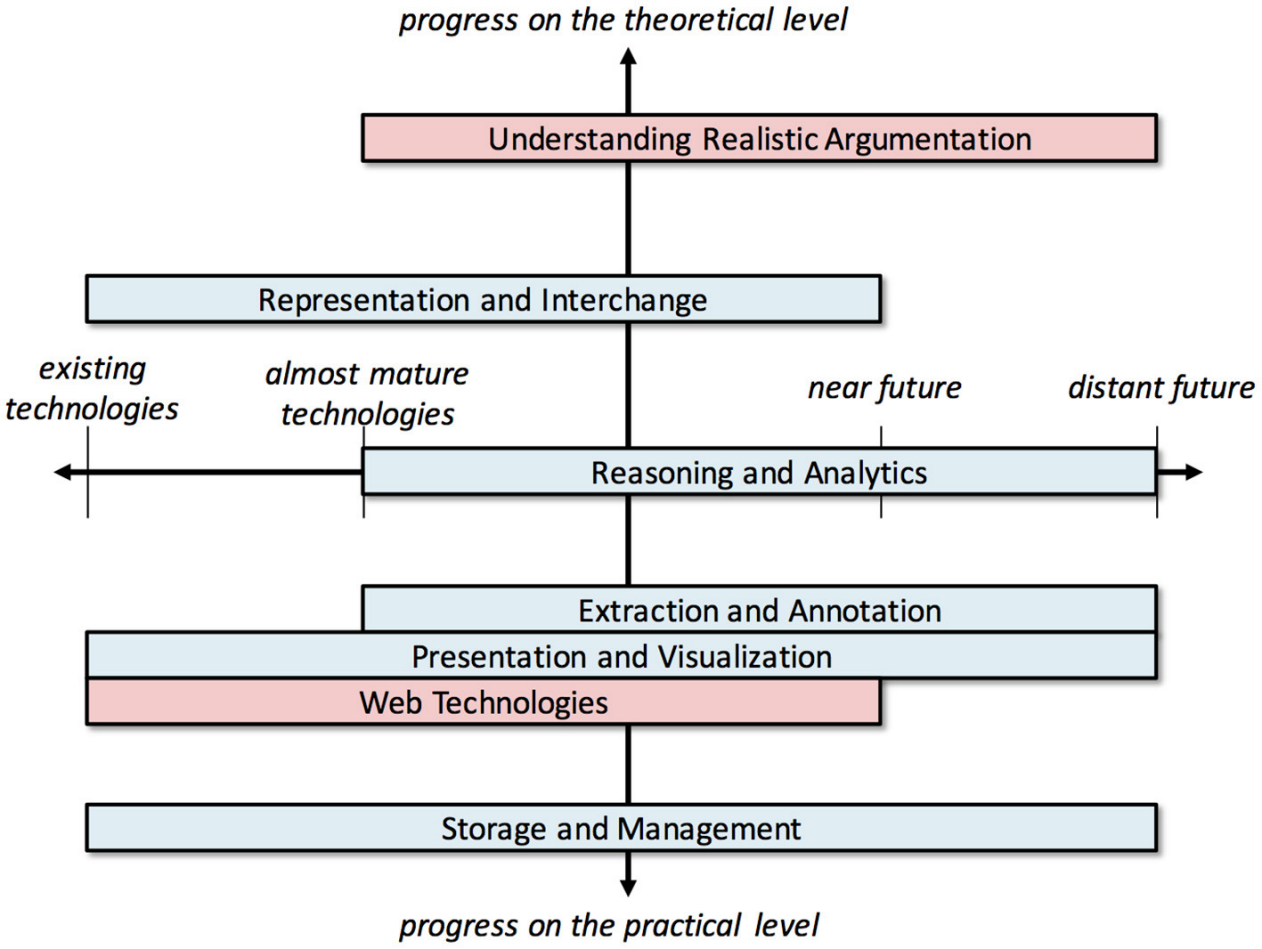
A 2-dimensional categorization of related technologies and challenges.

There are several research fields and state-of-the-art technologies that can provide the substrate upon which the vision of the Web of Debates can be realized, but also important obstacles that stand in the way of its realization. Figures 3, 4 provide an overview, showing some broad research fields and technologies that are relevant.

Figure 3 lists the main relevant technologies. The vertical bars represent various challenges that need to be overcome by the corresponding technologies and research fields. The horizontal bars represent critical technologies, which, even though not directly used to address any challenge, will set the guiding principles upon which the solutions to all challenges will be based. All these technologies need to be advanced or further explored to overcome the related challenges.

Figure 4 displays the same technologies and challenges from a different perspective, organizing them in a two-axis chart. The position of the technology along the horizontal axis represents both the current and the required maturity of each technology to solve the respective challenge. The left side of each rectangle represents the current capacity of the corresponding technology to address the related challenge, at least at a preliminary stage, whereas its right side represents additional advances that need to be achieved (and how far in the future these are estimated to occur) before actually solving the respective challenge in its entirety. On the other hand, the vertical axis represents the kind of progress required per technology (practical or theoretical) to overcome the respective challenge. We should note that this chart is based on our own assessment of the maturity level of each technology based on the literature we reviewed, and not on a systematic evaluation of the technologies.

In the following sections, we further analyse these technologies and their role in the realization of the Web of Debates.

### 4.1. Understanding realistic argumentation

*Argumentation theory* studies how conclusions can be reached through logical reasoning in the presence of, possibly contradictory, evidence for or against a certain conclusion, whereas *argumentation systems* are logic-based computational systems that aim to automate this process (see Baroni et al., 2018; Gabbay et al., 2021 for the state of the art and current trends). Scientific advances in these fields of study, such as the understanding of the structure of arguments, the development of tools for constructing arguments, the identification of their relations, and the development of semantics for drawing sound logical conclusions from possibly contradictory arguments, are all relevant in the context of the Web of Debates.

Nevertheless, the Web of Debates is a lot more than an argumentation system deployed in a global scale. The main challenge here is the shift from *logical argumentation* to *realistic argumentation*. Realistic argumentation does not only appeal to the logic of the audience, but also to its emotions. It is only partly based on facts and data, often employing additional techniques such as the clever use of verbal cues and the semantic structure of text/speech (politeness, aggressiveness etc), as well as different *argument schemes* based on factors such as appeal to authority or expert opinion, popularity of supported claims, peer-pressure, arguments from analogy, proof-by-example, non-logical (e.g., statistical) correlations between different arguments, and others (Walton, 2006). The aim of realistic argumentation is usually to *persuade* or *help reach a decision*, rather than prove or present facts or arguments for the sake of presenting them; thus, it also involves a process of selecting the strongest arguments to put forward first, taking into account their *relatedness, informativeness* or *persuasive* characteristics. In this sense, realistic argumentation is more context-aware and more personalized.

Building on the most influential model of arguments in the last decades, *Abstract Argumentation Frameworks* (Dung, 1995), there have been some attempts to formalize features of realistic argumentation, such as the audience (Hunter, 2015), the values that arguments promote (Bench-Capon, 2003), preferences (Amgoud and Vesic, 2014), trust (Villata et al., 2013), the argument strength (Amgoud et al., 2022), the context of argumentation (Brewka and Eiter, 2009), uncertain arguments (Hunter, 2013), commonsense arguments (Vassiliades et al., 2020), enthymemes (Black and Hunter, 2008), and persuasion dialogues (Prakken, 2009). There is also some promising research on the formalization of argumentation schemes (Verheij, 2003; Reed and Walton, 2005; Prakken et al., 2015; Wyner, 2016; Panisson et al., 2021), and more generally on the use of argumentation schemes in AI (Macagno, 2021). The study of what contributes to the persuasiveness or the quality an argument has recently started but is growing fast. Work on this topic includes crowdsourcing studies comparing arguments in terms of their persuasiveness (Habernal and Gurevych, 2016; Gretz et al., 2020), studies focusing on specific factors such as linguistic features (Persing and Ng, 2017), the semantic types (logos, ethos, or pathos) of claims and premises (Hidey et al., 2017), the types of evidence used to support an argument (Addawood and Bashir, 2016), personality traits and prior beliefs of the audience (Lukin et al., 2017; Durmus and Cardie, 2018; Al-Khatib et al., 2020), the style of the arguments (Baff et al., 2020), etc., but also some more general attempts to identify all related factors (Steenbergen et al., 2003; Wachsmuth et al., 2017a). According to Wachsmuth et al. (2017a), the quality of an argument is determined by its *cogency*, i.e., whether its premises are acceptable, relevant to its conclusion and sufficient to draw its conclusion; its *effectiveness*, which is related to the credibility of its author, its clarity, its emotional appeal and its appropriateness; and its *reasonableness*, which refers to its global acceptability, its relevance to the discussion or debate, and its ability to defend itself against all counter-arguments. The deliberative quality of an argument, defined in Steenbergen et al. (2003), includes additional factors that are important in deliberation dialogues, such as *respect, equality* among all arguers, *interactivity* and *testimoniality*. Most work in this area has the form of empirical studies aiming at validating the related factors, and improving our understanding of human argumentation. There are still, though, relevant issues from the perspective of discourse analysis, rhetorics, and psychology [e.g., whether people are skilled arguers (Hahn and Oaksford, 2012), and why people argue (Mercier and Sperber, 2011)] that has not yet attracted much attention from the AI community. Some other open research problems in this area concern the interaction of the different factors, how teams of arguments work in concert in debates, how the order that the arguments are presented influence the outcome of a debate, and how people select which arguments to put forward in a debate. The interdisciplinary study of such issues is necessary for understanding and formalizing human argumentation, which is in turn a key requirement for realizing the Web of Debates.

### 4.2. Web technologies

The current Web is based on the simple idea of interlinking documents and making them available to anyone from anywhere. Building on the same principle, different technologies have been proposed to extend the document Web. One of the most prominent ones is the *Semantic Web* (Berners-Lee et al., 2001) and the closely related *Linked Data* initiative, where the main building blocks are structured datasets (rather than documents). Its motivation is that documents are not easily machine-processable, so there are certain limitations on what a machine can do with them; on the other hand, access to machine-interpretable data (in the sense of a “global database”) can give rise to even more sophisticated applications, such as the ones that have already been created on top of knowledge graphs (see Hogan et al., 2021 for some examples).

The so-called *Social Web* aims to foster social interaction, by providing a plethora of tools and platforms enabling humans to communicate through blogging, tagging, Web content voting, social bookmarking, and other means of social interaction. The Web of Debates seeks to upgrade the role of the Social Web into a broader means of communicating opinions and carrying out debates. There have already been some attempts to integrate argumentation within the Social Web. For example, Schneider et al. (2013) provides a review of web applications that combine features of the Social Web, the Semantic Web and computational argumentation. Such applications, however, are still limited in the features of realistic argumentation they can support as they mostly rely on models that capture the logical aspects of argumentation. Frameworks for social argumentation (Leite and Martins, 2011; Baroni et al., 2015; Patkos et al., 2016) integrate arguments with social votes; online debates, though, involve a lot more non-logical aspects, which these frameworks do not capture. With a shift toward realistic arguments, knowledge exchange will be carried out along the lines of logical consistency, factual accuracy and some degree of emotional appeal to the intended audience, but will also take into account the individual needs and preferences of web users. Even though the decision of adopting one conclusion over another will remain a subjective issue, the Web of Debates will facilitate the process of deliberation by filtering out irrational and logically incorrect expressions, while maintaining a significant degree of personalization in choosing the top-rated arguments for each user.

The *Pragmatic Web* (Schoop et al., 2006) is motivated by the observation that the content of the Web does not actually represent factual data, but the subjective opinions of the people who upload it. Even though it has a similar motivation with the Web of Debates, its objectives and used methodologies are quite different. From the Pragmatic Web viewpoint, a conflict is just a clash of opinions, which is resolved not by analyzing the opinions themselves, but by determining the support of each opinion via crowdsourcing techniques, and by interpreting and representing data in a context-dependent manner so as to enable users to reach agreements. On the other hand, the Web of Debates aims to analyse and contrast the different contradicting arguments, to allow the interested user to better understand their connections, and eventually judge themselves the validity of each one, based on their own beliefs, knowledge, or even prejudice; unlike the approach followed by Pragmatic Web, this would allow the identification of widely spread, but unjustified, beliefs or opinions.

Closer to our vision is the *Argument Web* (Bex et al., 2013; Reed et al., 2017), which is an effort to deploy argumentation on the Web. At its core is the *Argument Interchange Format* (AIF, Chesñevar et al., 2006; Rahwan et al., 2007), an ontology for arguments. On top of AIF, several Web-based tools have been developed for argument annotation, visualization and analysis^7^ and have been applied to various types of real debates, including, for example, debates taking place in the famous BBC broadcast Moral Maze.^8^ Other applications include tools for better understanding existing arguments, or for improving the argumentation skills of adolescents.^9^ All these developments are in line with our vision of the Web of Debates and will contribute to its realization. These tools, however, rely mostly on manual annotation and analysis and cannot, therefore, meet the requirements of large-scale applications. The realization of the Web of Debates will require the automation of the argument annotation and analysis processes, their enhancement so that they can handle all features of human argumentation, and the development of several other extra-logical processes, such as profile and context analysis, audience analysis, trust analysis, reputation analysis and others. This will enable the development of large-scale web applications that can take advantage of all argumentative information that is already available on the Web.

In summary, the technological advances made in the context of the above technologies will contribute to the development of the Web of Debates in a critical manner. In particular, the low-level infrastructure of the Web of Debates is expected to reuse the standard Web protocols, whereas knowledge graph languages and semantic technologies, and other techniques and technologies such as crowdsourcing, social tagging, voting and others, which Web users are already familiar with, will probably find their way into the Web of Debates. The developments made in the Argument Web with respect to argument modeling, annotation and visualization will also be exploited and extended or adapted to the needs of the Web of Debates.

### 4.3. Extraction and annotation

As with all added-value technologies, the size of the Web of Debates must reach a critical mass to make itself useful. Given the abundance of the natural language arguments already on the Web, technologies such as automated mining of arguments from blogs, forums or other social media, Natural Language Processing (NLP) techniques and others, need to be employed to create structured arguments out of text. In addition, human contribution could be enabled for this task, by adapting existing technologies such as *gamification* (von Ahn and Dabbish, 2008) or *crowdsourcing* techniques. Some efforts have already been made to crowdsource argument creation (Chalaguine and Hunter, 2019) and annotation (Ghosh et al., 2014; Skeppstedt et al., 2018). Furthermore, aspects related to *multilinguality* should be addressed, exploiting the improving quality of automated translation tools. Along similar lines, the annotation of images, sounds or complete documents with the arguments that characterize them is equally critical for a Web where knowledge can take various forms.

In tandem with the above efforts, it is of crucial importance to encourage content providers to upload their arguments online using the proper format (i.e., in a structured form), by providing tools that simplify the process, e.g., by allowing the semi-automatic generation of arguments and/or by aiding the content provider annotate her arguments. Existing tools for manual argument creation or annotation, such as Araucaria (Reed and Rowe, 2004), Rationale (van Gelder, 2007), OVA (Bex et al., 2013), and Carneades (Gordon et al., 2007), enable the users to identify the components of arguments (e.g. their premises, conclusions, etc.), their relations (e.g., attack, support, etc.) and the argumentation schemes they instantiate (e.g., argument from expert opinion, etc.).

However, in order to be able to exploit the abundance of natural language arguments that already exist on the Web, automating the extraction of arguments from text is a fundamental requirement. The rapidly expanding field of *argument mining* (see Stede and Schneider, 2018; Lawrence and Reed, 2019 for a recent survey and book) has already demonstrated some promising results that could form the basis for realistic argument extraction and annotation in the Web of Debates. These include annotation schemes for argument mining (Budzynska and Reed, 2011; Peldszus and Stede, 2013; Stab and Gurevych, 2014; Kirschner et al., 2015; Habernal and Gurevych, 2017; Niculae et al., 2017), annotated corpora (Andreas et al., 2012; Ghosh et al., 2014; Rosenthal and McKeown, 2015; Abbott et al., 2016; Habernal and Gurevych, 2017), methods for argument extraction from text (Andreas et al., 2012; Florou et al., 2013; Ghosh et al., 2014; Rosenthal and McKeown, 2015; Abbott et al., 2016; Habernal and Gurevych, 2017) or for identification of argument relations (Peldszus and Stede, 2015; Cocarascu and Toni, 2017; Lawrence and Reed, 2017; Niculae et al., 2017; Nguyen and Litman, 2018; Kobbe et al., 2019; Trautmann et al., 2020). Most of the current corpora and argument mining methods have been developed for specific domains and applications and the performance varies across different tasks and domains; for example, the results are much better in persuasive essays (Stab and Gurevych, 2017) than in legal cases (Teruel et al., 2018) or microtexts (Peldszus and Stede, 2015), which are most commonly encountered on the Web. There is still lack of a general annotation scheme and generic methodologies that would perform well in multiple domains. We should note here that it may be impossible to develop a computational method that can with 100% accuracy identify arguments in a natural language text. As evidenced by several studies that involved manual annotation of texts (Stab and Gurevych, 2014; Kirschner et al., 2015; Habernal and Gurevych, 2017), there is very often disagreement between annotators on the arguments, components of arguments or argument relations conveyed by a text, which in most cases is due to the ambiguity of human language. As shown in Thorn Jakobsen et al. (2022), it may also be due to the different backgrounds and demographic characteristics of the annotators. Manual annotation may therefore introduce *social bias* to the data used to train data-driven argument mining methods and, as a result, also to the methods themselves. Addressing this challenge is a requirement for the realization of the Web of Debates, while methods for identifying and measuring biases (Pagano et al., 2023) can also help mitigate this issue. Most current argument mining approaches focus on arguments, components of arguments (e.g., premises and claim) or relations between arguments (e.g., attack and support). There have been some attempts to automatically extract from text other features of human argumentation such as ethotic expressions (Duthie and Budzynska, 2018), emotional arguments (Oraby et al., 2015) and argument schemes (Lawrence and Reed, 2016), but the research in this area is still in its early stages. Developing domain-independent methods with the capability of identifying extra-logical features of argumentation is essential for the development of solutions that better fit the needs of the Web of Debates.

### 4.4. Representation and interchange

Enabling the association and combination of arguments from different sites of the Web requires the development of a semantically explicit representation model (ontology) for realistic arguments, so that different independently developed applications will be able to process them in a common manner and interoperate within an integrated environment. As also discussed above, AIF (Chesñevar et al., 2006; Rahwan et al., 2007) is one such ontology, which captures various models of argument, both formal (such as AAFs), and informal such as Walton's *argumentation schemes* (Walton, 2006). Using AIF, it is possible to model the (logical) structure of an argument (e.g., its premises, conclusion, etc.), argument relations (e.g., support, conflict, preferences), but also the argumentation scheme that an argument adheres to. An extension of AIF enables also modeling elements of argumentative dialogues such as locutions (e.g., statements, withdrawals, questions, challenges, etc.), commitments and dialogue rules (Reed et al., 2008). Such approaches are definitely within the spirit of the Web of Debates. There are still though several aspects of human argumentation that have not been accommodated. The development of an appropriate model for realistic arguments requires answering additional questions such as: What are exactly the types of information that define the quality or persuasiveness of an argument? How are these modeled and attached to an argument? How do we characterize and model the presenter of an argument and her audience? What are the possible relations between realistic arguments and the possible statuses of an argument within a realistic debate? Most of these issues are still open research topics in computational argumentation, with some interesting approaches being proposed during the last few years (e.g., see Bench-Capon, 2012).

The representational model will be based on knowledge graph languages, to allow reusing existing ontologies that capture features related to realistic argumentation [e.g., profile ontologies such as UPOS (Sutterer et al., 2008) or provenance ontologies such as PROV-O (McGuinness et al., 2013)], and exploiting the Linked Open Data (LOD) architecture to provide connections between the concepts/topics related to the arguments and their representation in existing online datasets (e.g., Wikidata). This will enable interlinking related arguments, but also linking arguments with other types of web data, which can be used for example as supporting evidence. It will also allow using standard Semantic Web languages and tools (e.g., SPARQL, rule languages, etc.) for querying and reasoning with the arguments and their relationships.

### 4.5. Storage and management

Realistic arguments will be stored in what we call “*argument bases*”, the analogous of knowledge bases. Their structure will enable storing arguments, as well as any other information that is relevant to the proper representation of realistic arguments and debates. Argument bases should also provide: (*a*) inference support; (*b*) query support; (*c*) support for data management tasks such as updating, repairing and change monitoring; (*d*) alignment and interoperating capabilities with related ontologies; and (*e*) propagation of relevant information among different systems. For the development of such systems, the experience gained from the deployment of triple stores and other semantic data management systems (Özsu, 2016; Abdelaziz et al., 2017) will be exploited. The AIFdb database system (Lawrence et al., 2012), which was developed for storing and managing arguments described in the AIF ontology, supports some of the desired functionalities: it enables semantic processing and visualization of arguments, query management and dialogue control. A language for querying structured dialogical data, which is compatible with AIF and knowledge graph languages (RDF, SPARQL), was also recently developed (Zografistou et al., 2018). Such technologies are compatible with and can form the basis for the development of web-scale argument bases for the Web of Debates.

### 4.6. Reasoning and analytics

Representing and storing arguments in an adequate format is not an objective in itself, just the means toward providing adequate services over the Web of Debates, based on the general notions of analytics and reasoning. Through these services, the user will be able to search and navigate through arguments (possibly in an exploratory manner), pose structured queries over the pool of available arguments, or perform sophisticated (and customized) aggregation and summarization operations. In addition, sophisticated forms of reasoning may emerge, allowing the identification of implicit relationships among arguments, or the development of new forms of semantics that determine the “acceptability” of realistic arguments, along the tradition of abstract argumentation (Dung, 1995). There are already several tools, called *argumentation solvers*, that were designed to solve standard reasoning tasks (e.g., compute the set of acceptable arguments) in abstract argumentation frameworks—see Cerutti et al. (2017) for an overview and Lagniez et al. (2021) for the results of the latest International Competition on Computational Models of Argumentation. The standard acceptability semantics of AAFs, proposed in Dung (1995) and considered in all these tools, use two (*accepted*/*rejected*) or three values (*accepted*/*rejected*/*undetermined*) for representing the acceptability of arguments. This is, however, too simplistic compared to the way that we evaluate arguments in our every day life, where we most commonly believe in or are persuaded by arguments to varying degrees. This has recently led to finer-grain gradual evaluation methods, based on numerical scales (Baroni et al., 2019) or rankings (Bonzon et al., 2016). Some of these approaches also consider a *base weight*, a value assigned to an argument, which may represent the probability of believing the argument (Hunter, 2013), the aggregated strength of its premises and inference rules (Spaans, 2021), votes provided by users (Leite and Martins, 2011), the importance degree of a value promoted by the argument (Bench-Capon, 2003), or the trustworthiness of the argument's source (da Costa Pereira et al., 2011). Extending these methods to take into account the factors associated with the persuasiveness or quality of arguments discussed in Steenbergen et al. (2003) and Wachsmuth et al. (2017a) (see also Section 4.1) is a promising research direction that would contribute to the realization of the Web of Debates. A computational framework that combines an arbitrary set of factors to compute the overall quality or acceptance of an argument was proposed in Patkos et al. (2016); however, the framework is generic and takes only into account the users' arguments and votes. Further research is required to determine the extent to which each factor contributes to the quality of an argument, possible dependencies among the factors, and the role of the topic or context of a debate in determining which factors are more or less important.

Another aspect that should be taken into account is the much bigger scale of the Web of Debates compared to current argument-based applications. The majority of the reasoning problems in AAFs are known to be NP-hard (Charwat et al., 2015), and reasoning with realistic arguments is expected to be even more complex. The exact and complete solutions implemented by argumentation solvers may not, therefore, be feasible in scenarios involving large scale datasets. There have already been some recent efforts to develop approximate solutions for AAFs based on graph neural networks (Kuhlmann and Thimm, 2019; Craandijk and Bex, 2020; Malmqvist et al., 2020). The realization of the Web of Debates will require the development of similar approximate solutions for the evaluation of realistic arguments.

The *automated generation of arguments* on the basis of data or other arguments found on the Web will also be a desirable feature for many applications of the Web of Debates. This will create additional value from existing arguments, via aggregation, summarization, elaboration, and generation of new knowledge in the form of new realistic arguments. This is similar to how reasoning and inference generates new knowledge from existing facts based on well-defined formal deductive rules. In this direction, the approach proposed in Khatib et al. (2021), where arguments are generated by GPT-2, a neural language model, trained with data from argument knowledge graphs, has demonstrated promising results and a methodology that fits the envisioned features of the Web of Debates.

### 4.7. Presentation and visualization

Given the sheer size of the Web, one expects to find a large number of arguments in favor (or against) a certain claim, so presenting everything to the user is certainly not productive. Some kind of *aggregation* or *summarization* is necessary, along with a *ranking* process that will highlight the most important or relevant ones, taking into account also issues like the diversification of opinions. It should be emphasized that ranking only aims at the practical necessity to give priority to some of the arguments; the user should have access to all arguments, and no filtering or censorship should take place as part of the ranking process. Preliminary research in this area has focused on identifying similar arguments using clustering techniques (Misra et al., 2015; Boltuzic and Snajder, 2016) and on summarizing the key issues brought up in debates using standard text summarization techniques (Ranade et al., 2013), tools and techniques from lexical semantics (Saint-Dizier, 2018), or machine learning techniques and word embeddings (Misra et al., 2017).

A similar challenge is related to the *visualization* of arguments and their relationships, which is important for the content consumer to understand the structure of a complex web of realistic arguments. Tools such as Araucaria, Rationale, OVA, and Carneades (discussed in a previous section) visualize debates as trees or graphs, focusing on the logical part of arguments or their relationships. Other argument mapping tools are Kialo,^10^ which displays one argument at a time with its support arguments on one side and the attacking arguments on the other, and DebateGraph,^11^ which also focuses on one argument at a time and displays its related arguments in the form of a graph. Some of these tools display additional data about the arguments, such as a score or links to related debates or data. Such data but also any other information that is related to the quality or persuasiveness of an argument should be somehow made available to the users of the Web of the Debates and visualized in an intuitive way that will help them make sense of all different viewpoints in a debate as quickly as possible. Addressing the tradeoff between making available all relevant information to the users while, at the same time, helping them to make sense of a debate as quickly as possible is definitely a big challenge, and will require the adoption of standard information visualization principles such as the ones proposed by Shneiderman (1996), i.e., *overview, zoom and filter, details on demand, relate, history* and *export*.

## 5. Impact of the Web of Debates

### 5.1. Potential impact

The Web of Debates can be viewed as the “blog of tomorrow”, where people will be able not only to express their viewpoints in a natural language, but also to annotate and connect them in a machine-interpretable way. The expression of arguments in formal, machine-processable terms, as well as their interlinking, will create significant added-value benefits. In the same way that linked data and knowledge graphs have led to the discovery of new, previously unseen connections, correlations and knowledge (e.g., business analytics), we expect the interlinking of arguments to lead to a better understanding of the various debates and the generation of new, aggregated or previously unknown arguments and insights.

The abundance of Web data, combined with machine-processable arguments, will allow the envisioned version of the Web not only to provide relevant information (as when reading a book), but also to combine available data in order to provide arguments in favor of (or against) different alternative options (as done by a knowledgeable expert). This way, people will be better informed on matters of interest, thus promoting collective awareness on community problems and enabling better decision-making for professionals or companies.

At the community level, the services of the Web of Debates can enable public authorities to reach a broader audience in a more personalized way, in order to foster policies of societal value (e.g., healthy lifestyle, sound environmental behavior), to target unjustified concerns, to promote participation in community matters and democratic processes (e-democracy), or to support legal argumentation and justice attribution. At the individual level, the same services are expected to form a critical component of future autonomous entities endowed with socio-cognitive intelligence, which are used in the emerging market of smart spaces (Alazab et al., 2022). This can find applications ranging from service robots for domestic use, to smart environments related to domestic care and work, education, healthcare, communication and entertainment.

In addition, there is a wide range of potential applications suitable for the private sector; these generally fall under marketing, e.g., persuading customers to buy products/services, convincing people to donate to a charity, etc. Similarly, the Web of Debates can also be used as an assistive tool for individuals that practice persuasion as part of their professional life, such as lawyers, business executives etc, or for decision-makers in general, as it would allow better and more informed choices by combining information found on the Web, and also possibly in local databases, to build persuasive arguments and suggestions. But at the same time, by relying on transparent and easily configurable algorithms that promote the diversification of the viewpoints they present to the users, it can also help mitigate the problem of echo chambers and the increased polarization that this phenomenon causes.

Ultimately, we see the Web of Debates as the platform of ideas that holds the promise for promoting the role of humans in collective decision-making and e-democracy, able to have significant impact at both the individual and the societal level.

### 5.2. Ethical issues

The ability of the Web of Debates to adapt to the personal characteristics and background knowledge of each user requires that it has access to this information. However, it is important to ensure both that the users will be in total control of their personal data, and that the functionality of the Web of Debates will not be diminished by the lack of personal data. This can be ensured by developing the Web of Debates according to the *Privacy by Design* principles (Cavoukian, 2013). Following these principles, the Web of Debates should by default not have access to any personal data, its operations should be visible and transparent to all users, it should provide several data-sharing options that will be easily comprehensible to all users, and it should employ end-to-end security mechanisms for protecting the users' data.

We acknowledge the fact that persuasion (that underlies the Web of Debates), as well as the development of automated persuasion systems, would, by their very nature, be open for misuse by governments, businesses, individuals or organizations (e.g., for coercion, control or opinion enforcement). For example, one potential issue would be the usage of the Web of Debates as a means to promote the incorporation of false, deceptive or misleading arguments by malicious content providers. In both cases, naive content consumers could be deceived, thus causing disillusionment to well-intentioned users and jeopardizing the usefulness of the Web of Debates.

Despite the fact that such opportunities for abuse are admittedly present, this is the case for most useful technologies, so we argue that this should not be a deterring factor toward realizing this technology. As a most striking example, one could refer to today's Web, where all such features exist (inaccurate or false information, etc.). However, we argue that the Web of Debates will in fact improve the situation, and will be helpful toward mitigating this problem.

In particular, it should be noted that it is not the aim of the Web of Debates to provide any kind of censorship or checking on different opinions. On the contrary, it will allow all opinions to be more easily publishable and accessible. We argue that this feature will in fact reduce the opportunities for censorship, coercion, or deception, in the sense that access to different opinions, as well as the verification of the validity of arguments associated with these opinions, will be easier for open-minded content consumers, so the power of deceptive or misleading arguments and opinions will be mitigated.

Similarly, understanding persuasion (in general) can reduce the opportunities of coercion, control, or manipulation that may potentially be exercised by businesses, individuals or organizations over unaware citizens. Research on persuasion can help in identifying how and when this happens, as well as in preventing it, by allowing humans and intelligent systems to argue together.

At a more technical level, advances in the fields of trust and automated fact-checking,^12^ as well as the incorporation of provenance information in realistic arguments could help users in the task of identifying deceptive or misleading arguments. This is similar to how the current Web has allowed recent advances in technology where facts and statements can be more easily checked for validity against the vast amount of the information available on the Web, using fact-checkers.^13^

Furthermore, the integration of models and methods from Explainable Artificial Intelligence (Banerjee and Barnwal, 2023), especially in the processes that involve Machine Learning algorithms (e.g., argument mining or argument generation) will contribute to the transparency, interpretability and understandability of the outputs of the Web of Debates tools and applications and to the establishment of trust with their users. Computational argumentation has already proved to be a very useful tool for developing explainable systems (Vassiliades et al., 2021), while the recent launch of the International Workshop on Argumentation for Explainable AI^14^ shows that this is an active area of interest for researchers in computational argumentation. We, therefore, anticipate that their involvement in the design and development of the Web of Debates will ensure that it will function as an explainable system.

## 6. Conclusion

Not long ago, the problem of information overload attracted the attention of different scientific communities, fueled by the increasing number of people posting and accessing information on the Web; nowadays, the increasing amount of user-generated reviews, comments and arguments on the Web may lead to a similar problem, that of opinion overload. In this paper, we looked ahead to a future version of the Web, where this problem can be overcome by exploiting the structure of realistic arguments and understanding the arguers' intentions. After motivating and describing our vision, we identified its main challenges and proposed research and technological directions to its realization, which can be summarized in: understanding and formalizing realistic arguments and debates; developing methods and tools for automatically generating structured arguments (e.g., by extracting arguments from text); developing appropriate models for the representation and interchange of arguments; creating systems for their storage and management; developing methods for analyzing arguments and debates; developing models and methods for summarizing and visualizing arguments and debates; and augmenting Web technologies with the ability to automatically process online arguments by integrating the above research developments.

We strongly believe that the realization of this vision will stipulate research in a wide range of domains—scientific, academic and commercial—and can lead to the development of innovative human-centered applications that will revolutionize Web experience. Apart from its evident impact on the organization of argument and knowledge exchange on the Web, this effort opens up a way to serve a higher-level purpose: by enabling people to locate the valid rational arguments in the sea of opinions of questionable credibility, as well as those arguments that better support them, it will empower critical thinking and facilitate the active participation of humans in collective governance processes. Ultimately, we see the Web of Debates as the platform of ideas that holds the promise for promoting the role of humans in collective decision-making and e-democracy, able to have significant impact at both the individual and the societal level.

## Data availability statement

The original contributions presented in the study are included in the article/supplementary material, further inquiries can be directed to the corresponding author.

## Author contributions

AB, GF, TP, and DP contributed to the conception of the main ideas. AB, GF, and TP contributed to the review of the relevant literature. AB and GF contributed to the revision of the paper. All authors contributed to the first version of the paper and read and approved the submitted version.

## Footnotes

^1^ https://en.wikipedia.org/wiki/Web_2.0#Web_1.0

^2^ http://en.wikipedia.org/wiki/Web_2.0

^3^ https://en.wikipedia.org/wiki/Semantic_Web

^4^ https://lod-cloud.net/

^5^ https://en.wikipedia.org/wiki/Echo_chamber

^6^ https://en.wikipedia.org/wiki/Filter_bubble

^7^ https://arg-tech.org/index.php/research

^8^ https://www.newsweek.com/artificial-intelligence-argument-debate-752199

^9^ https://www.independent.co.uk/tech/artificial-intelligence-debate-argue-bbc-science-tech-research-a8118191.html

^10^ https://www.kialo.com/

^11^ https://debategraph.org/

^12^ http://en.wikipedia.org/wiki/Fact_checking

^13^ https://en.wikipedia.org/wiki/List_of_fact-checking_websites

^14^ https://people.cs.umu.se/tkampik/argxai/2022.html
